# Sustained hip flexion contracture after femoral lengthening in patients with achondroplasia

**DOI:** 10.1186/s12891-018-2344-8

**Published:** 2018-11-29

**Authors:** Mi Hyun Song, Tae-Jin Lee, Jong Hyeop Song, Hae-Ryong Song

**Affiliations:** Department of Orthopaedic Surgery and Institute for Rare Diseases, Korea University Medical Center, Guro Hospital, 148 Gurodong-ro, Guro-gu, Seoul, 152-703 Republic of Korea

**Keywords:** Hip, Flexion contracture, Achondroplasia, Femoral lengthening

## Abstract

**Background:**

Hip flexion contracture often occurs after femoral lengthening in patients with achondroplasia, but few studies have investigated its development in these patients. The purpose of this study was to analyze sustained hip flexion contracture in achondroplasia patients who underwent femoral lengthening and to identify contributing factors.

**Methods:**

This study included 34 patients with achondroplasia who underwent femoral lengthening (mean age at operation, 11.1 years). Sustained hip flexion was defined as flexion contracture lasting > 6 months postoperatively despite physiotherapy. Demographic data, spinopelvic parameters (pelvic incidence, pelvic tilt, sacral slope, lumbar lordosis, and sagittal vertical axis), and quantitative assessments of femoral lengthening were investigated. The associations among these factors and the development of sustained hip flexion contracture were assessed.

**Results:**

Sustained hip flexion contracture developed in 13 (38%) of 34 achondroplasia patients after femoral lengthening. Eight (62%) of these 13 patients concomitantly exhibited limitation of knee flexion. Excessive femoral lengthening (odds ratio [OR], 1.450; 95% confidence interval [CI], 1.064 to 1.975; *p* = 0.019) and forward sagittal vertical axis tilt (OR, 1.062; 95% CI, 1.001 to 1.127; *p* = 0.047) contributed to sustained hip flexion contracture.

**Conclusions:**

Sustained hip flexion contracture frequently occurs after femoral lengthening in achondroplasia patients. Both excessive femoral lengthening and preoperative forward SVA tilt may contribute to the development of sustained hip flexion contracture in these patients.

## Background

Achondroplasia is the most common skeletal dysplasia, with an incidence of one in every 30,000 live births annually [1]. Clinical features of achondroplasia are spinopelvic deformities, such as thoracolumbar kyphosis and lumbosacral hyperlordosis [2–4], and rhizomelic short stature [1, 5, 6]. Rhizomelic short stature in patients with achondroplasia is an appropriate indication for femoral lengthening to reduce functional impairment and improve quality of life [7, 8], because intramembranous ossification mechanism remains intact in achondroplasia.

However, excessive femoral lengthening can lead to complications, including contracture of adjacent joints, delayed callus formation and consolidation, and fracture of the regenerate bones [9, 10]. Hip flexion contracture is one of the most serious complications and frequently requires surgical treatment despite physiotherapy [7, 8, 11, 12]. Nevertheless, few studies have systematically analyzed the development of hip flexion contracture after femoral lengthening in achondroplasia. The purpose of this study was to analyze sustained hip flexion contracture after femoral lengthening in achondroplasia patients and to identify contributing factors.

## Methods

Patients who were diagnosed with achondroplasia and underwent simultaneous bilateral femoral lengthening at our institute from 2004 to 2015 were included. Patients who had undergone femoral lengthening at another institute before referral, underwent tibial lengthening only, had other medical co-morbidities (such as disorders related to bone metabolism and neuromuscular disorders), or had incomplete medical records and radiographs were excluded. A total of 34 patients were enrolled. The mean age at femoral lengthening was 11.1 years (6.8–21.5 years), and the patients were followed up for 5.0 years (2.0–11.1 years).

### Surgical technique

**Fig. 1:**
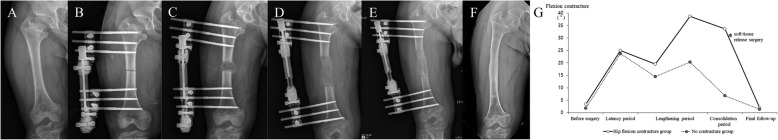
Radiographs of the different stages of the lengthening procedure and average hip flexion contracture at each measurement period. **a** Preoperative radiograph of a 16-year-old boy with achondroplasia **b**) Radiograph during the latency period of the patient. **c** Radiograph at the early stage of lengthening period of the patient. **d** Radiograph at the end of lengthening period of the patient. The patient finally gained the length 9.5 cm by femoral lengthening (lengthening percentage of 30.2%). **e** Radiograph during the consolidation period of the patient. **f** Radiograph after external fixator removal. The external fixator was removed after a bridging callus was observed at 3 of 4 cortices on plain radiographs. **g** Diagram of average hip flexion contracture for the entire patients during the each stage of the lengthening process. In the hip flexion contracture group, soft tissue release was performed during the consolidation period (*)

Femoral lengthening was performed by the senior surgeon (HRS) using a monolateral external fixator (Dyna-Extor, BK Meditech, Seoul, Korea) (Fig. 1). Before surgery, the patients were recommended performing limb lengthening of 30 to 40% of the initial segment length. Three Schanz screws were inserted into the proximal and distal ends of the femur perpendicular to the anatomic axis under fluoroscopic guidance. At the mid-diaphyseal level, a transverse osteotomy was performed with the multiple-drill-hole method. After 7 days of latent period, femoral lengthening was initiated at a rate of 0.25 mm every 6 h. The lengthening rate was adjusted according to the morphologic features of the callus [11, 12]. The external fixator was removed after a bridging callus was observed at 3 of 4 cortices on plain radiographs. A thigh brace was used for 4 to 6 weeks to prevent a regenerate fracture. Patients were followed up every week during the first month, every 2 weeks during the lengthening period, and then monthly during the consolidation period.

### Physiotherapy protocol

Passive range of motion (ROM) exercises of hip and knee joints were started on postoperative day 3. Simultaneously, the patients were encouraged to bear weight with the use of crutches. Active ROM exercises of adjacent joints were started on postoperative day 7. The patients were instructed to perform their exercise gently 3 times a day for 20 min. They were recommended to gradually increasing ROM and weight bearing as tolerated. In patients with restricted active ROM, passive and active-assisted ROM exercises were recommended priorly.

For patients with hip flexion contracture, hip extension exercise in the prone position was performed under a guidance of well-trained physician. The patients were instructed to perform their exercise 5 times a week at least 1 h, moreover, to be prone position on sleep.

### Outcome measurements

All patients measured their hip and knee ROM before surgery and at each period of the lengthening process. In addition, the existence of the hip flexion contracture was determined by the Thomas test [12, 13]. The Thomas test was performed with the patient in a supine position on a firm physical examination table. The uninvolved limb was adequately flexed to eliminate anterior pelvic tilt (PT) [14]. Concomitant knee joint stiffness was also assessed in every patient.

Patients were grouped into the hip flexion contracture group or the no significant contracture group based on the occurrence of sustained hip flexion contracture after femoral lengthening. Sustained hip flexion contracture was defined as a hip flexion contracture which lasted more than 6 months postoperatively despite intensive physiotherapy as well as which required soft tissue release eventually. All the soft tissue surgeries were performed during the consolidation period. For the soft tissue release, intramuscular recession of the rectus femoris [11] was mainly performed. Additionally, intramuscular recession of sartorius and iliopsoas and partial release of iliotibial band were often accompanied. Immediate postoperative hip flexion contractures, which were improved only with physiotherapy, were not considered to be sustained hip flexion contractures.

To identify potential contributors associated with sustained hip flexion contracture after femoral lengthening in achondroplasia, the following variables were investigated: sex, age at operation, preoperative spinopelvic parameters including pelvic incidence (PI), PT, sacral slope (SS), lumbar lordosis (LL), and sagittal vertical axis (SVA), and quantitative assessments of femoral lengthening.

**Fig. 2:**
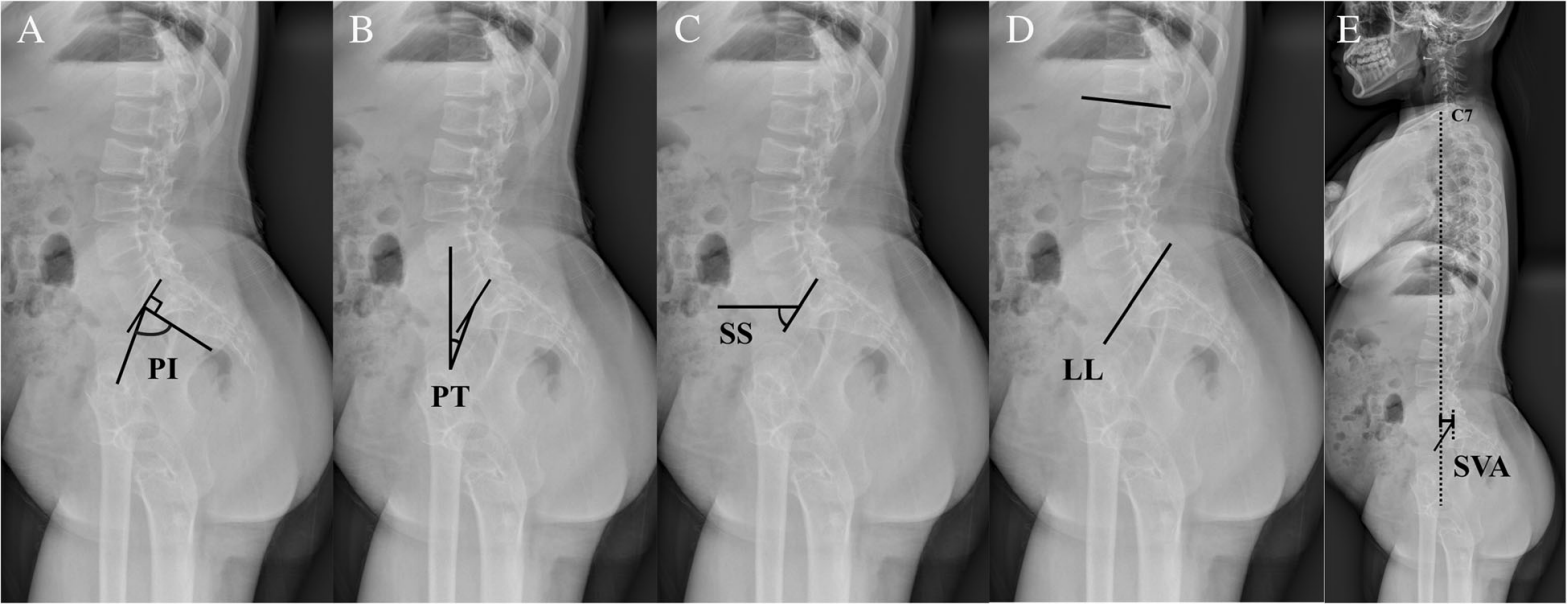
Spinopelvic parameters measured on standing radiographs. **a** Pelvic incidence (PI) was defined as the angle between a perpendicular line to the sacral endplate and a line joining the middle of the sacral endplate and the hip axis. **b** Pelvic tilt (PT) was defined as the angle between the line joining the middle of the sacral endplate and the hip axis and a vertical line. **c** Sacral slope (SS) was defined as the angle between the sacral superior endplate and the horizontal plane. **d** Lumbar lordosis (LL) was measured between the upper endplate of L1 and the upper endplate of S1 by using the Cobb method. **e** Sagittal vertical axis (SVA) was defined as the horizontal distance between a plumb line dropped from the center of C7 to the posterior-superior corner of S1

Spinopelvic parameters (PI, PT, SS, LL, and SVA) were measured on standing whole spine anteroposterior and lateral radiographs before femoral lengthening and at postoperative 6 months [2]. PI was defined as the angle between a perpendicular line to the sacral endplate and a line joining the middle of the sacral endplate and the hip axis (Fig. 2a), whereas PT was defined as the angle between the line joining the middle of the sacral endplate and the hip axis and a vertical line (Fig. 2b). SS was defined as the angle between the sacral superior endplate and the horizontal plane (Fig. 2c). LL was measured between the upper endplate of L1 and the upper endplate of S1 by using the Cobb method (Fig. 2d). SVA was defined as the horizontal distance between a plumb line dropped from the center of C7 to the posterior-superior corner of S1 (Fig. 2e).

Quantitative assessments of femoral lengthening were performed on a slit scanogram, as described by Bell and Thompson [15]. Initial femoral length, amount of lengthening, lengthening percentage, and external fixation index (EFI) were assessed. The lengthening percentage was defined as the amount of lengthening divided by the total length of the femur. The EFI was calculated as the duration of external fixation in days divided by the length gained in cm.

Besides, complications, except adjacent joints stiffness, were also assessed, according to the Paley’s classification [16]; which were subdivided into problem, obstacle, and sequel.

### Statistical analysis

Continuous data were analyzed with the Mann-Whitney test; and categorical data, with the Fisher exact test. Comparative analyses were performed with the Wilcoxon signed-rank test. Multivariate logistic regression analysis was performed to identify the potential contributors to sustained hip flexion contracture; dependent variables were those with *p*-values < 0.05 in univariate analysis or those with clinical significance. One side of the hip was randomly selected and statistically analyzed in all patients to avoid duplication of spinopelvic parameters. Statistical analyses were performed with SPSS version 21.0 (SPSS, IBM Corp., Chicago, IL); p-values < 0.05 were regarded as statistically significant.

## Results

Sustained hip flexion contracture occurred in 13 (38%) of 34 patients who underwent femoral lengthening. The patients in the hip flexion contracture group already had significantly larger degrees of hip flexion contracture before surgery (Table 1). However, the majority of patients of the hip flexion contracture group exhibited aggravation of hip flexion contracture by the end of lengthening period (*p* < 0.001) (Fig. 1). In spite of massive physiotherapy, unsolved hip flexion contracture remained in patients of the hip flexion contracture group; they required soft tissue release during the consolidation period. Eight (62%) of 13 patients with hip flexion contracture exhibited knee stiffness concomitantly, especially in limitation of flexion. After soft tissue surgeries, all patients of the hip flexion contracture group recovered nearly full range of movement of the hip and knee joint with no significant hip flexion contracture (Fig. 1).

**Table 1:** Comparison of the degree of hip flexion contracture between the hip flexion contracture and no significant contracture groups

Hip FC (°)	Hip FC group (*n* = 13)	No significant contracture group (*n* = 21)	*p*-value
Before surgery	6.3 (5–7)*	1.8 (0–5)*	**0.005†**
During the distraction osteogenesis			
Latency period	25.0 (10–35)*	23.7 (8–33)*	0.740
Lengthening period (early)	19.5 (0–25)*	14.5 (0–20)*	0.619
Lengthening period (end)	38.7 (32–42)*	20.3 (5–25)*	**< 0.001†**
Consolidation period	33.7 (30–35)*	6.8 (0–15)*	**< 0.001†**
Final follow-up	2.0 (0–5)*	1.3 (0–3)*	0.459

*FC* flexion contracture

*Data presented in parenthesis represent the range

†Statistically significant

The characteristics of the patients with hip flexion contracture are summarized in Table 2. These patients underwent larger amount and percentages of femoral lengthening (averagely 10.1 cm and 43.5%, respectively) compared to patients without contracture. In addition, they were more likely to show forward SVA tilt than patients without contracture.

**Table 2:** Comparative analyses of hip flexion contracture and no significant contracture groups

	Hip FC group (*n* = 13)	No significant contracture group (*n* = 21)	*p*-value
Demographic data			
Gender	4:9	8:13	0.665
Age at operation (years)	10.6 years (6.8–14.6)*	11.5 years (6.8–21.5)*	0.917
Preoperative spinopelvic parameters			
PI (°)	42.1 (36.3–50.2)*	35.6 (30.0–47.8)*	0.261
PT (°)	−2.5 (−6.7–12.2)*	− 3.6 (− 7.3–6.0)*	0.972
SS (°)	44.6 (37.4–55.6)*	39.1 (22.1–47.0)*	0.120
LL (°)	47.1 (37.2–54.3)*	43.2 (40.1–51.9)*	0.484
SVA (mm)	17.9 (−17–35.0)	−15.2 (− 47.0–40.0)	**0.005†**
Quantitative assessments of the femoral lengthening			
Initial femoral length	23.3 cm (20.2–26.2)*	25.2 cm (18.0–40.0)*	0.649
Amount of lengthening	10.1 cm (7.6–13.6)*	8.1 cm (5.8–10.9)*	**0.001†**
Lengthening percentage	43.5% (36.5–52.1)*	33.3% (19.0–45.6)*	**< 0.001†**
EFI (day/cm)	35.7 (21.8–56.6)*	41.4 (23.3–60.0)*	0.309

*FC* flexion contracture, *PT* pelvic tilt, *PI*, pelvic incidence, *SS* sacral slope, *LL* lumbar lordosis, *EFI* external fixation index

*Data presented in parenthesis represent the range

†Statistically significant

The amount of lengthening and the preoperative SVA value were significantly associated with sustained hip flexion contracture. Excessive femoral lengthening increased the risk of sustained hip flexion contracture (odds ratio [OR], 1.450; 95% confidence interval [CI], 1.064 to 1.975; *p* = 0.019). In addition, as the preoperative SVA shifted forwards, the risk of sustained hip flexion contracture increased (OR, 1.062; 95% CI, 1.001 to 1.127; *p* = 0.047).

**Fig. 3:**
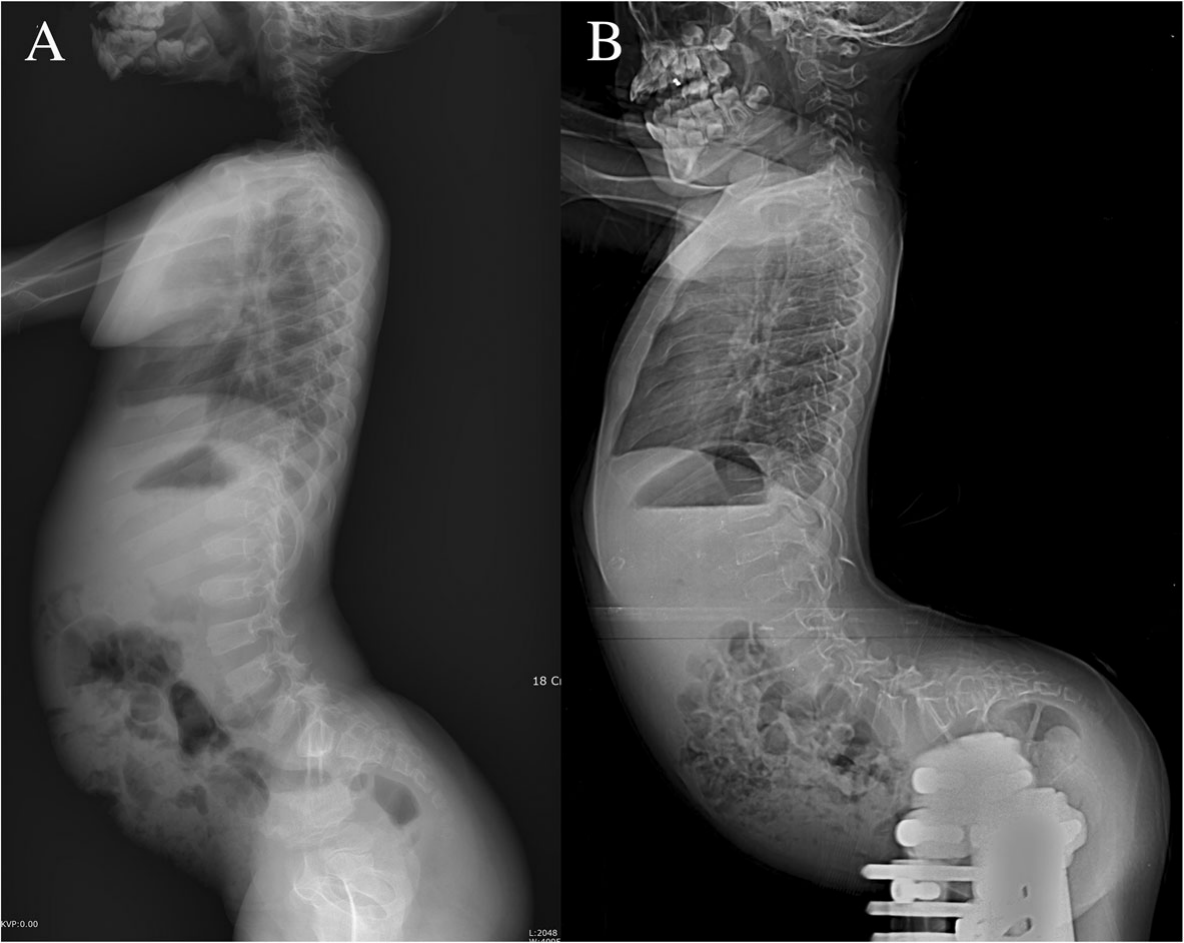
Radiographs of a 10-year-old boy with achondroplasia exhibiting sustained hip flexion contracture after femoral lengthening. **a** Initial radiograph of the patient. The patient underwent simultaneous bilateral femoral lengthening to gain 11 cm (lengthening percentage of 42.9%). **b** Postoperative radiograph showing an aggravation of the horizontal sacrum with lumbosacral hyperlordosis

**Fig. 4:**
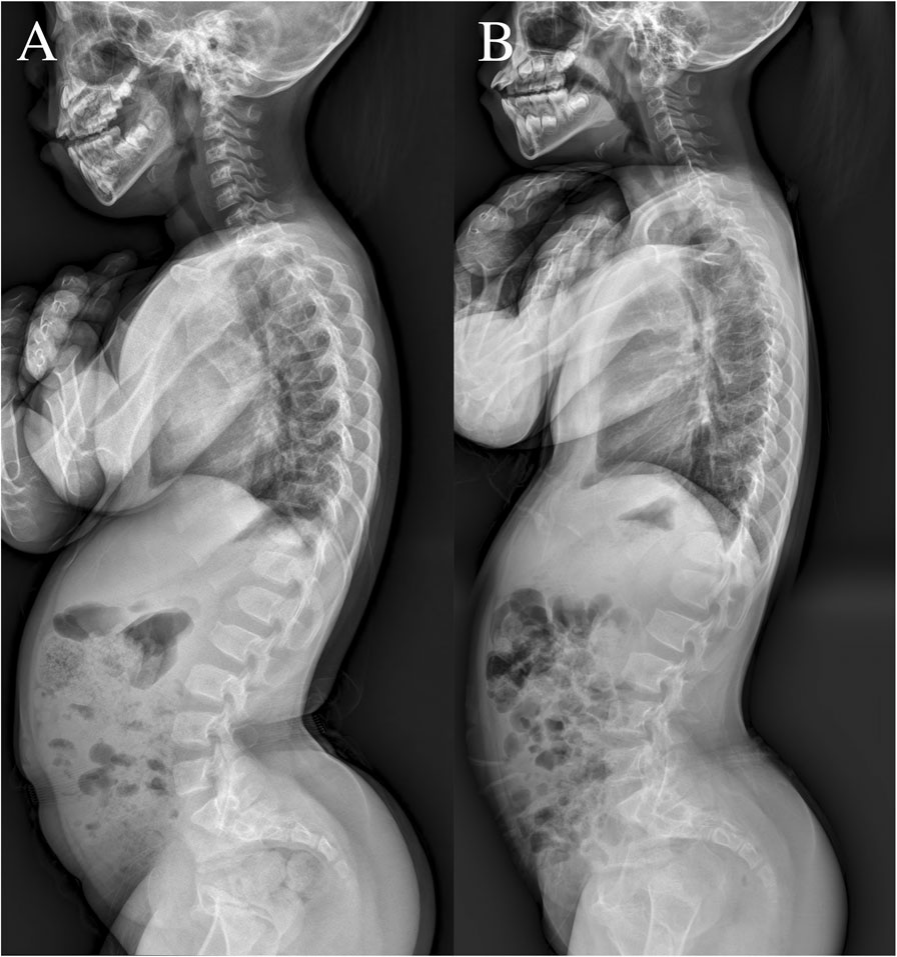
Radiographs of a 16-year-old achondroplasia patient not exhibiting flexion contracture after femoral lengthening. **a** Initial radiograph of the patient. The patient underwent simultaneous bilateral femoral lengthening to gain 6 cm (lengthening percentage of 19.0%). **b** Postoperative radiograph showing no significant changes of spinopelvic parameters in comparison with initial radiograph

Perioperative spinopelvic parameters between hip flexion contracture and no significant contracture groups were in Table 3. In the hip flexion contracture group, a significant change in spinopelvic parameters developed during the femoral lengthening (Fig. 3): LL and SS were significantly increased (*p* = 0.001 and 0.001, respectively) postoperatively, whereas PT was decreased (*p* = 0.011). There were no significant interval changes in PI. In contrast, no significant interval changes in spinopelvic parameters developed in the no significant contracture group (Fig. 4).

**Table 3:** Perioperative spinopelvic parameters between hip flexion contracture and no significant contracture groups

	Hip FC group (n = 13)			No significant contracture group (n = 21)		
	Preoperative	postoperative	*p*-value	preoperative	postoperative	*p*-value
PI (°)	42.1 (36.3–50.2)*	53.3 (29.2–65.5)	0.068	35.6 (30.0–47.8)*	36.0 (28.5–52.1)	0.317
PT (°)	−2.5 (−6.7–12.2)*	− 5.0 (− 26.0–24.0)	**0.011†**	− 3.6 (− 7.3–6.0)*	− 3.0 (− 26.0–17.0)	0.317
SS (°)	44.6 (37.4–55.6)*	74.3 (45.0–107.0)	**0.001†**	39.1 (22.1–47.0)*	40.0 (20.0–72.0)	0.919
LL (°)	47.1 (37.2–54.3)*	66.7 (45.0–87.0)	**0.001†**	43.2 (40.1–51.9)*	48.0 (40.8–66.0)	0.281

*FC* flexion contracture, PT, pelvic tilt, *PI* pelvic incidence, *SS* sacral slope, *LL* lumbar lordosis

*Data presented in parenthesis represent the range

†Statistically significant

Complications of the hip flexion contracture and no significant contracture groups were as follows. In the hip flexion contracture group, refracture developed in 8 patients after external fixator removal. They underwent external fixator reapplication using an intramedullary nailing with or without bone grafting. Two patients had varus angular deformity and underwent osteotomy for acute correction. One patient exhibited superficial pin site infection which required treatment with oral antibiotics. In contrast, 7 cases of refracture and 1 case of varus angular deformity developed in the no significant contracture group; the treatment method of each complication was same as that of the hip flexion contracture group. Additionally, one patient had deep surgical site infection. Local debridement was done in the operation room. There were no cases of limb length discrepancy at postoperation in both groups.

## Discussion

Patients with achondroplasia have unique pelvic and spinal structures. They exhibit a pelvis that is shaped like a champagne glass and has an anterior PT [17]. With regards to the spine, kyphosis at the thoracolumbar junction is very frequent in infants and mostly resolves when the child begins to walk [2–4]. As kyphosis begins to improve, lumbosacral hyperlordosis may begin to progress [17]. Hence, the spinopelvic parameters of patients with achondroplasia differ from those of the average population: they are more likely to have higher thoracolumbar kyphosis and LL as well as lower PT and thoracic kyphosis [2–4]. As a result, patients with achondroplasia are at greater risk of developing hip flexion contracture than the average population. Indeed, we previously demonstrated that hip flexion contracture often occurs after femoral lengthening in achondroplasia patients [11, 12].

We found that excessive femoral lengthening was a significant contributor to sustained hip flexion contracture after femoral lengthening in achondroplasia patients. This result is consistent with previous reports. Many authors have recommended lengthening the bony segment from 20 to 30% of its initial length to prevent complications [10, 18–20]. We previously demonstrated that adjacent joint contracture is more common when femoral lengthening is greater than 50% in achondroplasia patients [12]. This occurs because excessive distraction osteogenesis beyond muscle elasticity induces substantial irreversible muscle damage with fibrosis, inflammation, and necrosis [20–22]. In the present study, femoral osteotomy was performed in the mid-diaphyseal region, so damage could have arisen in the quadriceps and hamstring muscles [23, 24].

Hong et al. [2] demonstrated that pelvic parameters (PI, SS, and PT) are closely related to spinal parameters (thoracic kyphosis, LL, and thoracolumbar kyphosis) in achondroplasia patients, and that these relationships play an important role in achieving sagittal balance. Besides, the trunk of achondroplasia patients tends to backward tilt (mean SVA, − 22.2 mm) to compensate for lumbar hyperlordosis [25]. However, the present study indicated that preoperative forward SVA tilt is another contributor to sustained postoperative hip flexion contracture. The authors postulated the reasons that patients who have preoperative forward SVA tilt might have be at greater risk of pre-existing hip flexion contracture and quadriceps shortening before femoral lengthening.

Postoperatively, patients with sustained hip flexion contracture showed a significant change in spinopelvic parameters, including an increase in SS and LL and a decrease in PT. In addition, most patients had knee stiffness, especially in limitation of flexion. These findings suggest that the quadriceps muscles, mainly the rectus femoris, may be the main origin of the hip flexion contracture. Tightness and stiffness of the quadriceps muscles induce an imbalance of muscle force around the pelvis and compensatory anterior pelvic tilt, which sequentially leads to a significant increase in SS and lumbar hyperlordosis. Moreover, this may eventually cause deterioration of perioperative hip flexion contracture in these patients [25]. This could explain why all patients having sustained hip flexion contracture underwent soft tissue release, including intramuscular recession of the rectus femoris, and recovered near full range of motion of the hip joint as a consequence.

This study had several limitations. First, the sample size was not large enough. Second, hip flexion contracture was confirmed by the Thomas test only, instead of accompanying the gait analysis. These limitations were inevitable due to the rarity of achondroplasia and the retrospective study design. Nevertheless, all patients in our series were surgically treated by a single surgeon at a single institution with a long-term follow-up. In addition, it is important to note that this study is the first attempt to analyze the sustained hip flexion contracture after femoral lengthening in achondroplasia patients.

## Conclusions

Sustained hip flexion contracture is frequent after femoral lengthening in achondroplasia patients. Both excessive femoral lengthening and preoperative forward SVA tilt may contribute to the development of sustained hip flexion contracture in these patients. It would be better for surgeons to consider preemptive soft tissue surgery during femoral lengthening in achondroplasia patients of these situations.

## Abbreviations

EFI: external fixation index
LL: lumbar lordosis
PI: pelvic incidence
PT: pelvic tilt
SS: sacral slope
SVA: sagittal vertical axis
